# The genome sequence of a greenbottle fly, *Lucilia caesar* (Linnaeus, 1758) (Diptera: Calliphoridae)

**DOI:** 10.12688/wellcomeopenres.26152.1

**Published:** 2026-03-17

**Authors:** Steven Falk, Liam M. Crowley, Olga Sivell, University of Oxford and Wytham Woods Genome Acquisition Lab, Natural History Museum Genome Acquisition Lab, Darwin Tree of Life Barcoding Collective, Wellcome Sanger Institute Tree of Life Management, Samples and Laboratory team, Wellcome Sanger Institute Scientific Operations: Sequencing Operations, Wellcome Sanger Institute Tree of Life Core Informatics team, Tree of Life Core Informatics collective, Darwin Tree of Life Consortium

**Affiliations:** 1 Independent researcher, Kenilworth, Warwickshire, England, UK; 2 University of Oxford, Oxford, England, UK; 3 Natural History Museum, London, England, UK

**Keywords:** Lucilia caesar; greenbottle fly; genome sequence; chromosomal; Diptera

## Abstract

We present a genome assembly from an individual male *Lucilia caesar* (greenbottle fly; Arthropoda; Insecta; Diptera; Calliphoridae). The genome sequence has a total length of 615.55 megabases. Most of the assembly (88.01%) is scaffolded into 6 chromosomal pseudomolecules, including the X sex chromosome. The mitochondrial genome has also been assembled, with a length of 15.94 kilobases. This assembly was generated as part of the Darwin Tree of Life project, which produces reference genomes for eukaryotic species found in Britain and Ireland.

## Species taxonomy

Eukaryota; Opisthokonta; Metazoa; Eumetazoa; Bilateria; Protostomia; Ecdysozoa; Panarthropoda; Arthropoda; Mandibulata; Pancrustacea; Hexapoda; Insecta; Dicondylia; Pterygota; Neoptera; Endopterygota; Diptera; Brachycera; Muscomorpha; Eremoneura; Cyclorrhapha; Schizophora; Calyptratae; Oestroidea; Calliphoridae; Luciliinae; *Lucilia; Lucilia caesar* (Linnaeus, 1758) (NCBI:txid65466).

## Background

*Lucilia caesar* (Linnaeus, 1758) is a metallic green fly from the family Calliphoridae (blow flies). This species can be difficult to separate from other *Lucilia*, particularly *Lucilia illustris* and *Lucilia ampullacea.* What distinguishes *L. caesar* from *L. ampullacea* is the presence of a coxopleural streak and 2 to 3 irregular rows of black hairs on the occiput (hairs are mainly pale in *L. ampullacea*). The hairs on the occiput of *L. illustris* are mostly black. The frons of *L. caesar* is very narrow, while *L. illustris* can be distinguished by wider frons, almost as wide as a single parafacial. Male and female genitalia of *Lucilia* species are distinctive. ( Draber-Mońko, 2004; Rognes, 1991; Sivell *et al.*, 2019; Sivell, 2021; van Emden, 1954). The keys to larval stages were provided by Szpila *et al.* (2013) (first instar) and Szpila (2009) (third instar).

Molecular identification of *L. caesar* using the COI barcode can pose difficulties due to its similarity with *L. illustris*; additional genetic markers, e.g. ND6 and ND5 show promising results, but further research is needed ( Schoofs *et al.*, 2018; Sonet *et al.*, 2012).

*Lucilia caesar* is widely distributed in the Palaearctic ( Draber-Mońko, 2004; Rognes, 1991). It is one of the most common and widespread greenbottles in Britain ( MacLeod, 1943, 1963; MacLeod & Donnelly, 1956; Sivell, 2021) The adults are on the wing from April to October ( Sivell, 2021). This species is synanthropic.

The adults feed on carrion, faeces, ripe fruit and on flowers aiding pollination. They are also attracted to the stinkhorn fungus *Phallus impudicus* Linnaeus, 1753. The females lay eggs on carrion and larvae are predominantly saprophagous. *Lucilia caesar* is a species of forensic importance. According to Matuszewski *et al.* (2011), it is the earliest coloniser of large carrion in forests of Central Europe in summer.

The fly may also oviposit on a live host, particularly one that is wounded or soiled. The larvae feed on its tissues in a condition called myiasis which, when untreated, may lead to host’s death. *L. caesar* was reported in cases of myiasis in sheep *,* cows, hedgehogs *,* wild boar, roe deer, rabbits, domestic cats and dogs ( Haddow & Thomson, 1937; MacLeod, 1943; Martínez-Calabuig *et al.*, 2023; Morris & Titchener, 1997; Pezzi *et al.*, 2021; Principato & Cioffi, 1996; Wetzel & Fischer, 1971). *L. caesar* was also reported in cases of myiasis in humans ( Bernhardt *et al.*, 2018; Franza *et al.*, 2006; Rognes, 1991). According to Baer (1931) *L. caesar* has been used in larval therapy for healing wounds. The larvae are used as fish bait and may cause allergic reactions in some people ( Siracusa *et al.*, 1989, 1994).

Despite its importance for forensic, medical and veterinary entomology there is relatively little data on the development of this common blow fly. Some observations have been made by Nuorteva (1977).

The chromosomally complete genome sequence for *Lucilia caesar* has been generated as part of the Darwin Tree of Life project, a collaborative effort to sequence all named eukaryotic species in the Atlantic Archipelago of Britain and Ireland. It will aid research on the phylogeny of Calliphoridae and taxonomy, biology, and ecology of the species.

## Methods

### Sample acquisition and DNA barcoding

**Figure 1.**
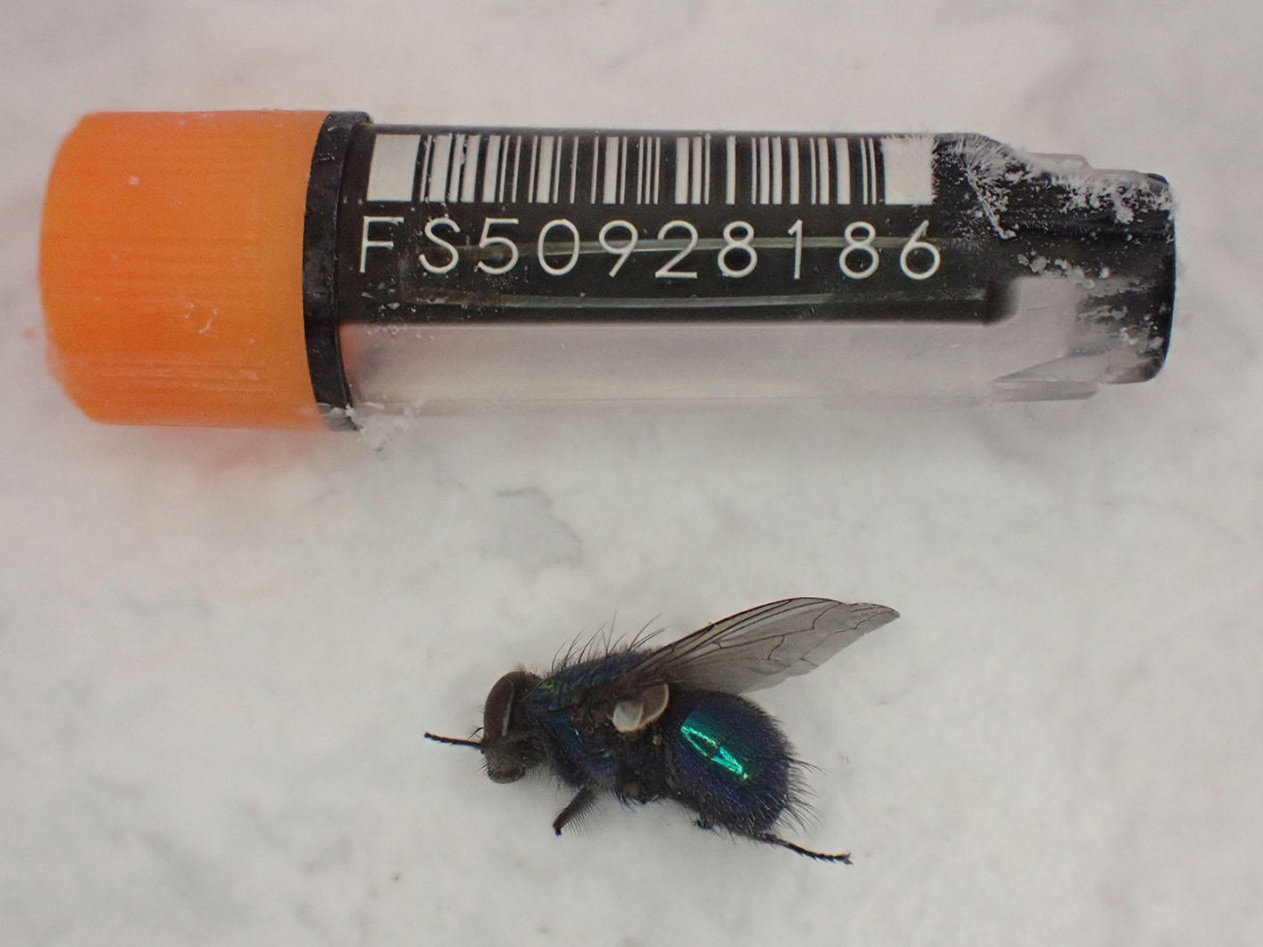
Photograph of the *Lucilia caesar* (idLucCaes4) specimen used for genome sequencing.

The specimen used for genome sequencing was an adult male *Lucilia caesar* (specimen ID Ox004085, ToLID idLucCaes4; Figure 1), collected from Cothill Fen, Oxforshire, UK (latitude 51.695, longitude −1.335) on 2023-05-23. The specimen was collected by Liam Crowley and Steven Falk and identified by Steven Falk. A second specimen was used for Hi-C sequencing (specimen ID Ox000753, ToLID idLucCaes1). It was collected from Wytham Woods, Oxfordshire, United Kingdom (latitude 51.77, longitude −1.331) on 2020-08-04. The specimen was collected and identified by Steven Falk. A third specimen was used for RNA sequencing (specimen ID NHMUK014444470, ToLID idLucCaes2). It was collected from Hever Castle, England, UK (latitude 51.188, longitude 0.12) on 2020-08-27. The specimen was collected and identified by Olga Sivell.

The initial identification was verified by an additional DNA barcoding process according to the framework developed by Twyford *et al.* (2024). A small sample was dissected from the specimen and stored in ethanol, while the remaining parts were shipped on dry ice to the Wellcome Sanger Institute (WSI) (see the protocol). The tissue was lysed, the COI marker region was amplified by PCR, and amplicons were sequenced and compared to the BOLD database, confirming the species identification ( Crowley *et al.*, 2023). Following whole genome sequence generation, the relevant DNA barcode region was also used alongside the initial barcoding data for sample tracking at the WSI ( Twyford *et al.*, 2024). The standard operating procedures for Darwin Tree of Life barcoding are available on protocols.io.

### Nucleic acid extraction

Protocols for high molecular weight (HMW) DNA extraction developed at the Wellcome Sanger Institute (WSI) Tree of Life Core Laboratory are available on protocols.io ( Howard *et al.*, 2025). The idLucCaes4 sample was weighed and triaged to determine the appropriate extraction protocol. Tissue from the whole organism was homogenised by powermashing using a PowerMasher II tissue disruptor. HMW DNA was extracted using the Manual MagAttract v3 protocol. DNA was sheared into an average fragment size of 12–20 kb following the Megaruptor®3 for LI PacBio protocol. Sheared DNA was purified by automated SPRI (solid-phase reversible immobilisation). The concentration of the sheared and purified DNA was assessed using a Nanodrop spectrophotometer and Qubit Fluorometer using the Qubit dsDNA High Sensitivity Assay kit. Fragment size distribution was evaluated by running the sample on the FemtoPulse system. For this sample, the final post-shearing DNA had a Qubit concentration of 40.6 ng/μL and a yield of 5 278.00 ng.

RNA was extracted from abdomen tissue of idLucCaes2 in the Tree of Life Laboratory at the WSI using the RNA Extraction: Automated MagMax™ *mir*Vana protocol. The RNA concentration was assessed using a Nanodrop spectrophotometer and a Qubit Fluorometer using the Qubit RNA Broad-Range Assay kit. Analysis of the integrity of the RNA was done using the Agilent RNA 6000 Pico Kit and Eukaryotic Total RNA assay.

### PacBio HiFi library preparation and sequencing

Library preparation and sequencing were performed at the WSI Scientific Operations core. Libraries were prepared using the SMRTbell Prep Kit 3.0 (Pacific Biosciences, California, USA), following the manufacturer’s instructions. The kit includes reagents for end repair/A-tailing, adapter ligation, post-ligation SMRTbell bead clean-up, and nuclease treatment. Size selection and clean-up were performed using diluted AMPure PB beads (Pacific Biosciences). DNA concentration was quantified using a Qubit Fluorometer v4.0 (ThermoFisher Scientific) and the Qubit 1X dsDNA HS assay kit. Final library fragment size was assessed with the Agilent Femto Pulse Automated Pulsed Field CE Instrument (Agilent Technologies) using the gDNA 55 kb BAC analysis kit.

The sample was sequenced on a Revio instrument (Pacific Biosciences). The prepared library was normalised to 2 nM, and 15 μL was used for making complexes. Primers were annealed and polymerases bound to generate circularised complexes, following the manufacturer’s instructions. Complexes were purified using 1.2X SMRTbell beads, then diluted to the Revio loading concentration (200–300 pM) and spiked with a Revio sequencing internal control. The sample was sequenced on a Revio 25 M SMRT cell. The SMRT Link software (Pacific Biosciences), a web-based workflow manager, was used to configure and monitor the run and to carry out primary and secondary data analysis.

### Hi-C

***Sample preparation and crosslinking***

The Hi-C sample was prepared from 20–50 mg of frozen head tissue from the idLucCaes1 sample using the Arima-HiC v2 kit (Arima Genomics). Following the manufacturer’s instructions, tissue was fixed and DNA crosslinked using TC buffer to a final formaldehyde concentration of 2%. The tissue was homogenised using the Diagnocine Power Masher-II. Crosslinked DNA was digested with a restriction enzyme master mix, biotinylated, and ligated. Clean-up was performed with SPRISelect beads before library preparation. DNA concentration was measured with the Qubit Fluorometer (Thermo Fisher Scientific) and Qubit HS Assay Kit. The biotinylation percentage was estimated using the Arima-HiC v2 QC beads.

***Hi-C library preparation and sequencing***

Biotinylated DNA constructs were fragmented using a Covaris E220 sonicator and size selected to 400–600 bp using SPRISelect beads. DNA was enriched with Arima-HiC v2 kit Enrichment beads. End repair, A-tailing, and adapter ligation were carried out with the NEBNext Ultra II DNA Library Prep Kit (New England Biolabs), following a modified protocol where library preparation occurs while DNA remains bound to the Enrichment beads. Library amplification was performed using KAPA HiFi HotStart mix and a custom Unique Dual Index (UDI) barcode set (Integrated DNA Technologies). Depending on sample concentration and biotinylation percentage determined at the crosslinking stage, libraries were amplified with 10–16 PCR cycles. Post-PCR clean-up was performed with SPRISelect beads. Libraries were quantified using the AccuClear Ultra High Sensitivity dsDNA Standards Assay Kit (Biotium) and a FLUOstar Omega plate reader (BMG Labtech).

Prior to sequencing, libraries were normalised to 10 ng/μL. Normalised libraries were quantified again to create equimolar and/or weighted 2.8 nM pools. Pool concentrations were checked using the Agilent 4200 TapeStation (Agilent) with High Sensitivity D500 reagents before sequencing. Sequencing was performed using paired-end 150 bp reads on the Illumina NovaSeq 6000.

### RNA library preparation and sequencing

Libraries were prepared using the NEBNext® Ultra™ II Directional RNA Library Prep Kit for Illumina (New England Biolabs), following the manufacturer’s instructions. Poly(A) mRNA in the total RNA solution was isolated using oligo (dT) beads, converted to cDNA, and uniquely indexed; 14 PCR cycles were performed. Libraries were size-selected to produce fragments between 100–300 bp. Libraries were quantified, normalised, pooled to a final concentration of 2.8 nM, and diluted to 150 pM for loading. Sequencing was carried out on the Illumina NovaSeq 6000, generating paired-end reads.

### Genome assembly

Prior to assembly of the PacBio HiFi reads, a database of *k*-mer counts ( *k* = 31) was generated from the filtered reads using FastK. GenomeScope2 ( Ranallo-Benavidez *et al.*, 2020) was used to analyse the *k*-mer frequency distributions, providing estimates of genome size, heterozygosity, and repeat content.

The HiFi reads were assembled using Hifiasm ( Cheng *et al.*, 2021) with the --primary option. The Hi-C reads ( Rao *et al.*, 2014) were mapped to the primary contigs using bwa-mem2 ( Vasimuddin *et al.*, 2019), and the contigs were scaffolded in YaHS ( Zhou *et al.*, 2023) with the --break option for handling potential misassemblies. The scaffolded assemblies were evaluated using Gfastats ( Formenti *et al.*, 2022), BUSCO ( Manni *et al.*, 2021) and MerquryFK ( Rhie *et al.*, 2020).

The mitochondrial genome was assembled using MitoHiFi ( Uliano-Silva *et al.*, 2023).

### Assembly curation

The assembly was decontaminated using the Assembly Screen for Cobionts and Contaminants ( ASCC) pipeline. TreeVal was used to generate the flat files and maps for use in curation. Manual curation was conducted primarily in PretextView and HiGlass ( Kerpedjiev *et al.*, 2018). Scaffolds were visually inspected and corrected as described by Howe *et al*. (2021). Manual corrections included 524 breaks and 802 joins. This reduced the scaffold count by 11.2%, reduced the scaffold N50 by 17.4%, and reduced the total assembly length by 7.9%. The curation process is described at https://gitlab.com/wtsi-grit/rapid-curation . PretextSnapshot was used to generate a Hi-C contact map of the final assembly.

### Assembly quality assessment

The MerquryFK tool ( Rhie *et al.*, 2020) was run in a Singularity container ( Kurtzer *et al.*, 2017) to evaluate *k*-mer completeness and assembly quality for the primary and alternate haplotypes using the *k*-mer databases ( *k* = 31) computed prior to genome assembly. The analysis outputs included assembly QV scores and completeness statistics.

The genome was analysed using the BlobToolKit pipeline, a Nextflow implementation of the earlier Snakemake version ( Challis *et al.*, 2020). The pipeline aligns PacBio reads using minimap2 ( Li, 2018) and SAMtools ( Danecek *et al.*, 2021) to generate coverage tracks. It runs BUSCO ( Manni *et al.*, 2021) using lineages identified from the NCBI Taxonomy ( Schoch *et al.*, 2020). For the three domain-level lineages, BUSCO genes are aligned to the UniProt Reference Proteomes database ( Bateman *et al.*, 2023) using DIAMOND blastp ( Buchfink *et al.*, 2021). The genome is divided into chunks based on the density of BUSCO genes from the closest taxonomic lineage, and each chunk is aligned to the UniProt Reference Proteomes database with DIAMOND blastx. Sequences without hits are chunked using seqtk and aligned to the NT database with blastn ( Altschul *et al.*, 1990). The BlobToolKit suite consolidates all outputs into a blobdir for visualisation. The BlobToolKit pipeline was developed using nf-core tooling ( Ewels *et al.*, 2020) and MultiQC ( Ewels *et al.*, 2016), with containerisation through Docker ( Merkel, 2014) and Singularity ( Kurtzer *et al.*, 2017).

## Genome sequence report

### Sequence data

**Figure 2.**
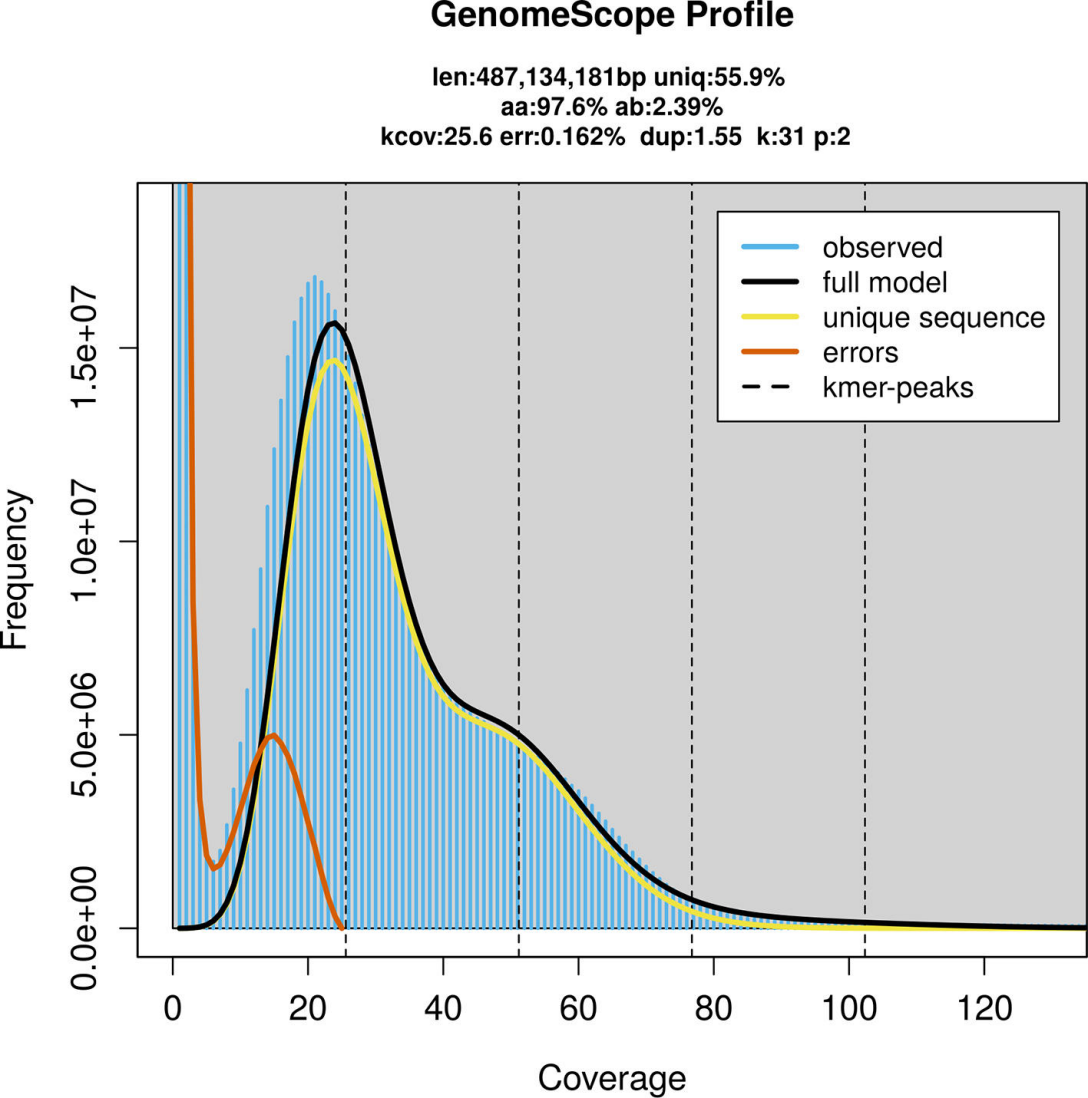
Frequency distribution of *k*-mers generated using GenomeScope2. The plot shows observed and modelled *k*-mer spectra, providing estimates of genome size, heterozygosity, and repeat content based on unassembled sequencing reads.

PacBio sequencing of the *Lucilia caesar* specimen generated 26.33 Gb (gigabases) from 3.83 million reads, which were used to assemble the genome. GenomeScope2.0 analysis estimated the haploid genome size at 489.16 Mb, with a heterozygosity of 2.39% and repeat content of 44.15% ( Figure 2). These estimates guided expectations for the assembly. Based on the estimated genome size, the sequencing data provided approximately 51× coverage. Hi-C sequencing produced 118.57 Gb from 785.22 million reads, which were used to scaffold the assembly. RNA sequencing data were also generated and are available in public sequence repositories. Table 1 summarises the specimen and sequencing details.

**Table 1.** Specimen and sequencing data for BioProject PRJEB83539.

Platform	PacBio HiFi	Hi-C	RNA-seq
**ToLID**	idLucCaes4	idLucCaes1	idLucCaes2
**Specimen ID**	Ox004085	Ox000753	NHMUK014444470
**BioSample (source individual)**	SAMEA114645007	SAMEA7746466	SAMEA8534295
**BioSample (tissue)**	SAMEA114645694	SAMEA7746545	SAMEA8534301
**Tissue**	whole organism	head	abdomen
**Instrument**	Revio	Illumina NovaSeq 6000	Illumina NovaSeq 6000
**Run accessions**	ERR14106134	ERR14075566	ERR14075571
**Read count total**	3.83 million	785.22 million	69.13 million
**Base count total**	26.33 Gb	118.57 Gb	10.44 Gb

### Assembly statistics

The primary haplotype was assembled, and contigs corresponding to an alternate haplotype were also deposited in INSDC databases. The final assembly has a total length of 615.55 Mb in 857 scaffolds, with 1 541 gaps, and a scaffold N50 of 104.11 Mb ( Table 2).

**Table 2.** Genome assembly statistics.

**Assembly name**	idLucCaes4.1
**Assembly accession**	GCA_965655125.1
**Alternate haplotype accession**	GCA_965655155.1
**Assembly level**	chromosome
**Span (Mb)**	615.55
**Number of chromosomes**	6
**Number of contigs**	2 398
**Contig N50**	0.63 Mb
**Number of scaffolds**	857
**Scaffold N50**	104.11 Mb
**Sex chromosomes**	X
**Organelles**	Mitochondrion: 15.94 kb

**Figure 3.**
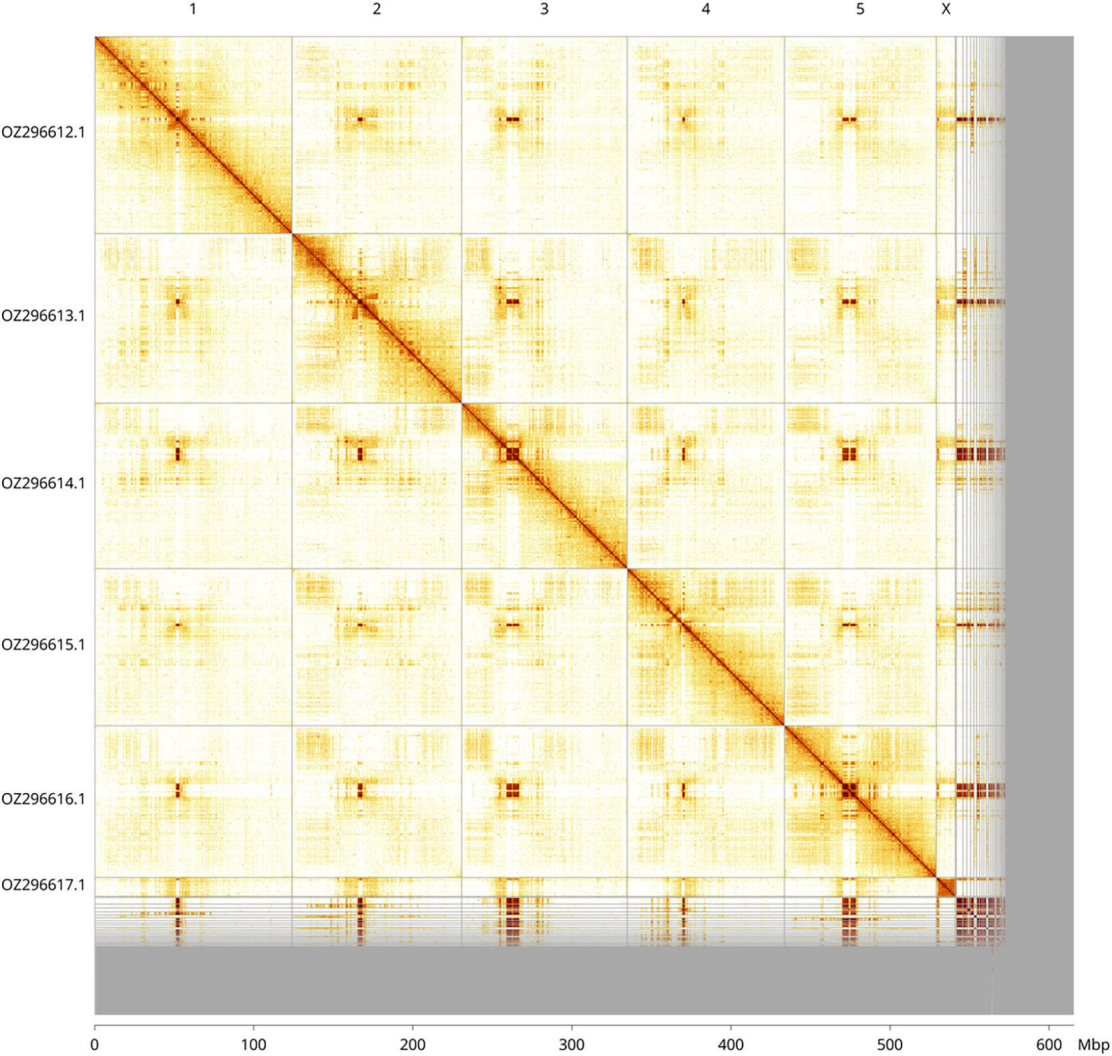
Hi-C contact map of the *Lucilia caesar* genome assembly. Assembled chromosomes are shown in order of size and labelled along the axes, with a megabase scale shown below. The plot was generated using PretextSnapshot.

Most of the assembly sequence (88.01%) was assigned to 6 chromosomal-level scaffolds, representing 5 autosomes and the X sex chromosome. These chromosome-level scaffolds, confirmed by Hi-C data, are named according to size ( Figure 3; Table 3). The X chromosome was identified by read coverage, but no Y chromosome was found. Males of closely related species are known to have the X0 karyotype. A large number of centromeric repeats is in the unlocalised sequences.

**Table 3.** Chromosomal pseudomolecules in the primary genome assembly of *Lucilia caesar* idLucCaes4.

INSDC accession	Molecule	Length (Mb)	GC%
OZ296612.1	1	124.12	29.50
OZ296613.1	2	106.71	30.50
OZ296614.1	3	104.11	30
OZ296615.1	4	98.80	30.50
OZ296616.1	5	95.38	30.50
OZ296617.1	X	12.63	30.50

The mitochondrial genome was also assembled (length 15.94 kb, OZ296618.1). This sequence is included as a contig in the multifasta file of the genome submission and as a standalone record.

### Assembly quality metrics

**Figure 4.**
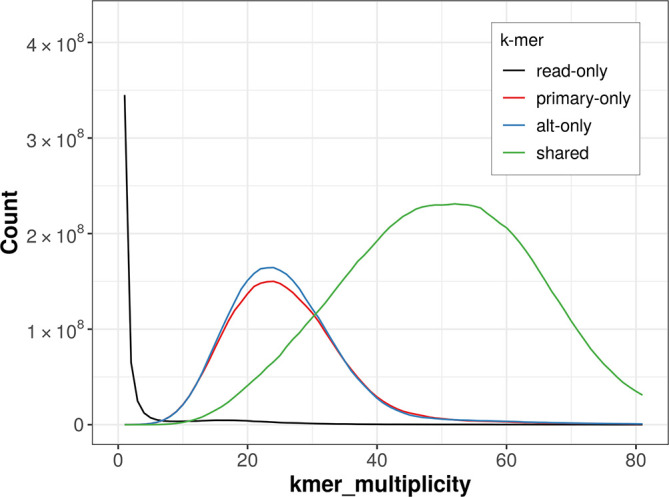
Evaluation of *k*-mer completeness using MerquryFK. This plot illustrates the recovery of *k*-mers from the original read data in the final assemblies. The horizontal axis represents *k*-mer multiplicity, and the vertical axis shows the number of *k*-mers. The black curve represents *k*-mers that appear in the reads but are not assembled. The green curve corresponds to *k*-mers shared by both haplotypes, and the red and blue curves show *k*-mers found only in one of the haplotypes.

The combined primary and alternate assemblies achieve an estimated QV of 52.5. The *k*-mer completeness is 70.27% for the primary assembly, 71.84% for the alternate haplotype, and 98.82% for the combined assemblies ( Figure 4).

**Figure 5.**
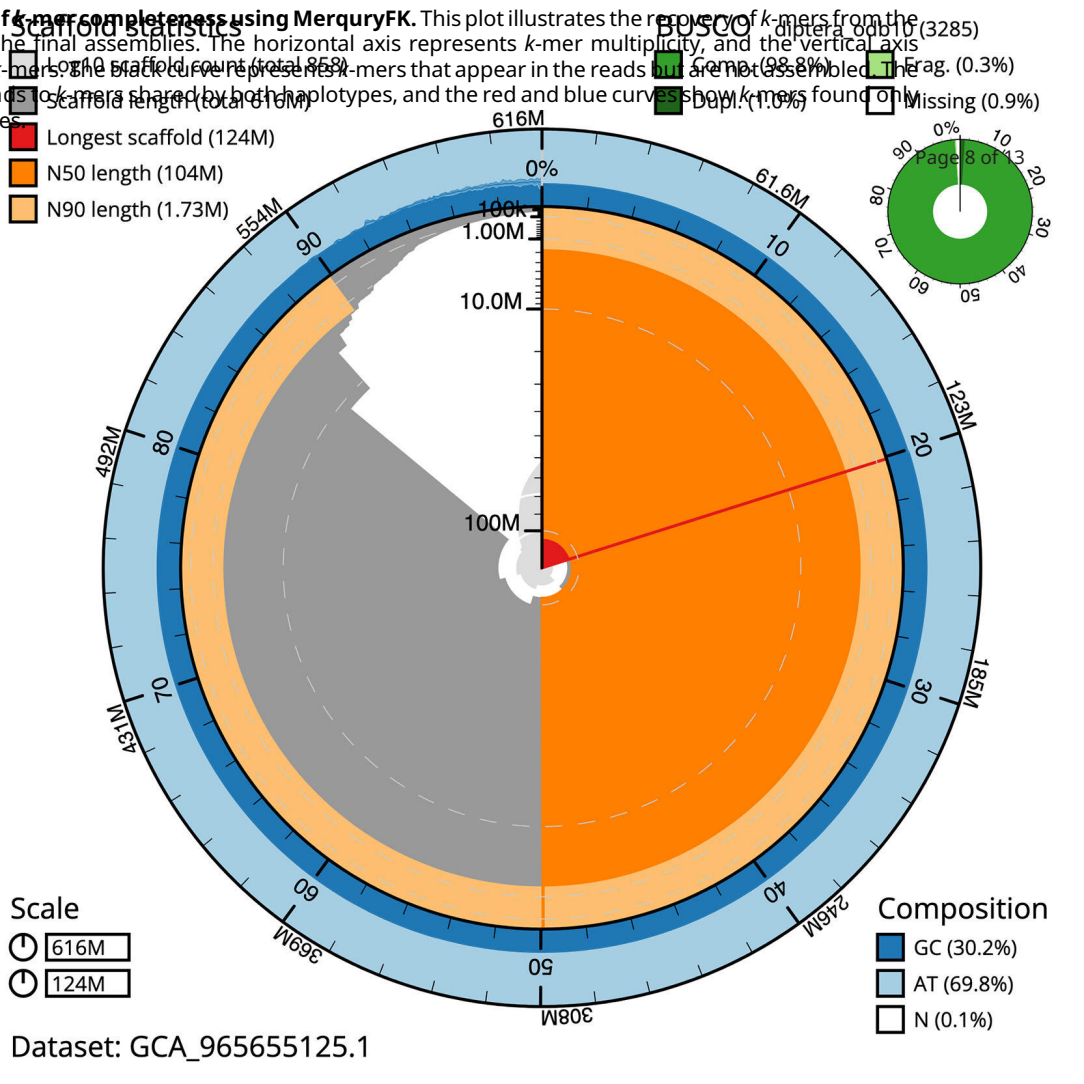
Assembly metrics for idLucCaes4.1. The BlobToolKit snail plot provides an overview of assembly metrics and BUSCO gene completeness. The circumference represents the length of the whole genome sequence, and the main plot is divided into 1 000 bins around the circumference. The outermost blue tracks display the distribution of GC, AT, and N percentages across the bins. Scaffolds are arranged clockwise from longest to shortest and are depicted in dark grey. The longest scaffold is indicated by the red arc, and the deeper orange and pale orange arcs represent the N50 and N90 lengths. A light grey spiral at the centre shows the cumulative scaffold count on a logarithmic scale. A summary of complete, fragmented, duplicated, and missing BUSCO genes in the endopterygota_odb10 set is presented at the top right. An interactive version of this figure can be accessed on the BlobToolKit viewer.

**Figure 6.**
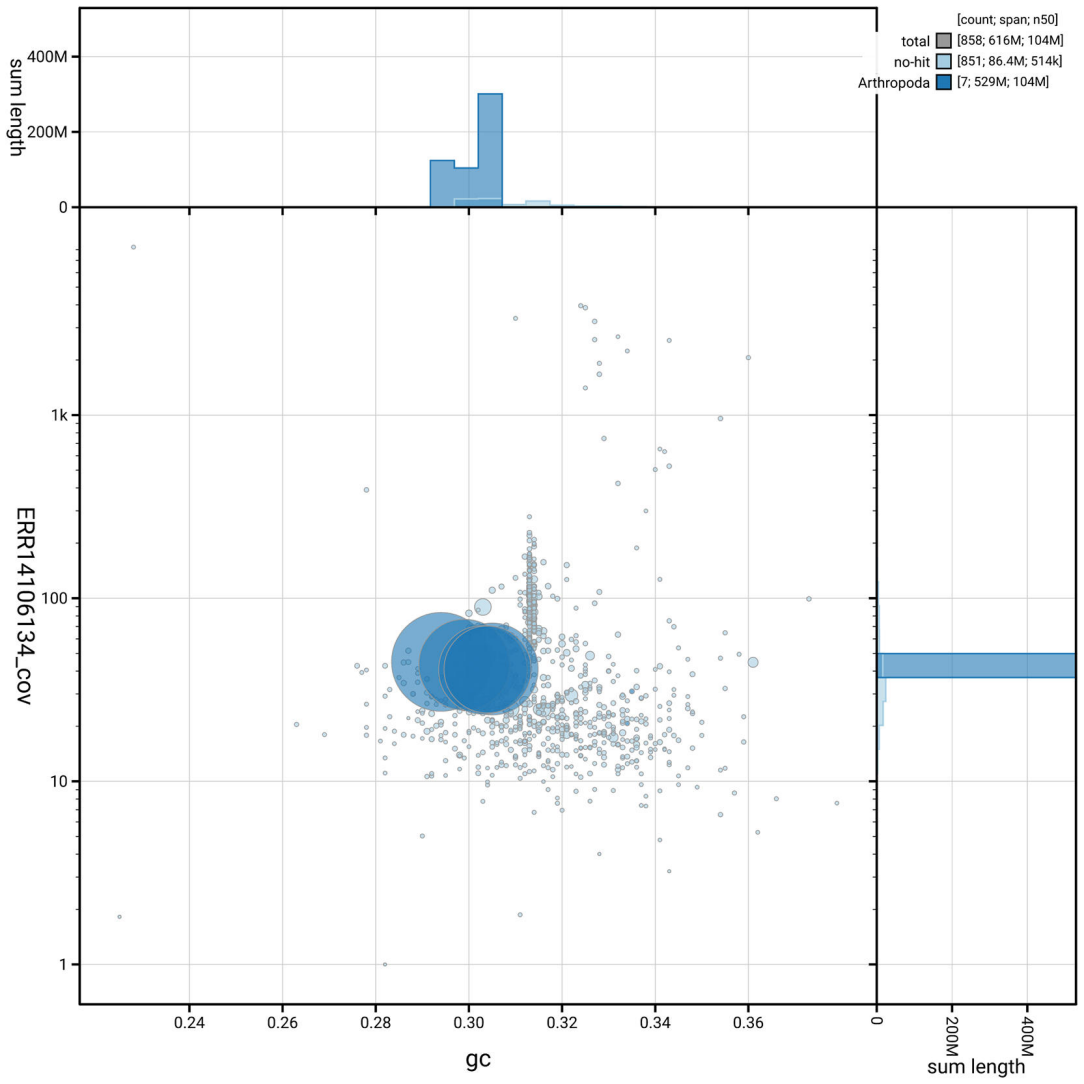
BlobToolKit blob plot for idLucCaes4.1. The plot shows base coverage (vertical axis) and GC content (horizontal axis). The circles represent scaffolds, with the size proportional to scaffold length and the colour representing phylum membership. The histograms along the axes display the total length of sequences distributed across different levels of coverage and GC content. An interactive version of this figure is available on the BlobToolKit viewer.

BUSCO v.6.0.0 analysis using the endopterygota_odb10 reference set ( *n* = 2 124) identified 99.2% of the expected gene set (single = 98.0%, duplicated = 1.2%). The snail plot in Figure 5 summarises the scaffold length distribution and other assembly statistics for the primary assembly. The blob plot in Figure 6 shows the distribution of scaffolds by GC proportion and coverage.

Table 4 lists the assembly metric benchmarks adapted from Rhie *et al.* (2021) and the Earth BioGenome Project Report on Assembly Standards September 2024. The EBP metric, calculated for the primary assembly, is **5.8.Q52**.

**Table 4.** Earth biogenome project summary metrics for the *Lucilia caesar* assembly.

Measure	Value	Benchmark
EBP summary (primary)	5.8.Q52	6.C.Q40
Contig N50 length	0.63 Mb	≥ 1 Mb
Scaffold N50 length	104.11 Mb	= chromosome N50
Consensus quality (QV)	Primary: 52.3; alternate: 52.6; combined: 52.5	≥ 40
*k*-mer completeness	Primary: 70.27%; alternate: 71.84%; combined: 98.82%	≥ 95%
BUSCO	C:99.2% [S:98.0%, D:1.2%], F:0.2%, M:0.6%, n:2 124	S > 90%; D < 5%
Percentage of assembly assigned to chromosomes	88.01%	≥ 90%

**Notes:** The EBP summary uses log10(Contig N50); chromosome-level (C) or log10(Scaffold N50); Q (Merqury QV). BUSCO: C = complete; S = single-copy; D = duplicated; F = fragmented; M = missing; n = orthologues

**Table 5.** Software versions and sources.

Software	Version	Source
BLAST	2.14.0	ftp://ftp.ncbi.nlm.nih.gov/blast/executables/blast+/
BlobToolKit	4.4.6	https://github.com/blobtoolkit/blobtoolkit
BUSCO	6.0.0	https://gitlab.com/ezlab/busco
bwa-mem2	2.2.1	https://github.com/bwa-mem2/bwa-mem2
DIAMOND	2.1.8	https://github.com/bbuchfink/diamond
fasta_windows	0.2.4	https://github.com/tolkit/fasta_windows
FastK	1.1	https://github.com/thegenemyers/FASTK
GenomeScope2.0	2.0.1	https://github.com/tbenavi1/genomescope2.0
Gfastats	1.3.6	https://github.com/vgl-hub/gfastats
Hifiasm	0.19.8-r603	https://github.com/chhylp123/hifiasm
HiGlass	1.13.4	https://github.com/higlass/higlass
MerquryFK	1.1.2	https://github.com/thegenemyers/MERQURY.FK
Minimap2	2.28-r1209	https://github.com/lh3/minimap2
MitoHiFi	3	https://github.com/marcelauliano/MitoHiFi
MultiQC	1.14; 1.17 and 1.18	https://github.com/MultiQC/MultiQC
Nextflow	24.10.4	https://github.com/nextflow-io/nextflow
PretextSnapshot	0.0.5	https://github.com/sanger-tol/PretextSnapshot
PretextView	1.0.3	https://github.com/sanger-tol/PretextView
samtools	1.21	https://github.com/samtools/samtools
sanger-tol/ascc	0.1.0	https://github.com/sanger-tol/ascc
sanger-tol/blobtoolkit	v0.9.0	https://github.com/sanger-tol/blobtoolkit
sanger-tol/curationpretext	1.4.2	https://github.com/sanger-tol/curationpretext
Seqtk	1.3	https://github.com/lh3/seqtk
Singularity	3.9.0	https://github.com/sylabs/singularity
TreeVal	1.4.0	https://github.com/sanger-tol/treeval
YaHS	1.2.2	https://github.com/c-zhou/yahs

## Author information

Contributors are listed at the following links:

• Members of the University of Oxford and Wytham Woods Genome Acquisition Lab
• Members of the Natural History Museum Genome Acquisition Lab
• Members of the Marine Biological Association Genome Acquisition Lab
• Members of the Darwin Tree of Life Barcoding collective
• Members of the Wellcome Sanger Institute Tree of Life Management, Samples and Laboratory team
• Members of Wellcome Sanger Institute Scientific Operations – Sequencing Operations
• Members of the Wellcome Sanger Institute Tree of Life Core Informatics team
• Members of the Tree of Life Core Informatics collective
• Members of the Darwin Tree of Life Consortium

## Wellcome sanger institute – Legal and governance

The materials that have contributed to this genome note have been supplied by a Darwin Tree of Life Partner. The submission of materials by a Darwin Tree of Life Partner is subject to the **‘Darwin Tree of Life Project Sampling Code of Practice’**, which can be found in full on the Darwin Tree of Life website. By agreeing with and signing up to the Sampling Code of Practice, the Darwin Tree of Life Partner agrees they will meet the legal and ethical requirements and standards set out within this document in respect of all samples acquired for, and supplied to, the Darwin Tree of Life Project. Further, the Wellcome Sanger Institute employs a process whereby due diligence is carried out proportionate to the nature of the materials themselves, and the circumstances under which they have been/are to be collected and provided for use. The purpose of this is to address and mitigate any potential legal and/or ethical implications of receipt and use of the materials as part of the research project, and to ensure that in doing so we align with best practice wherever possible. The overarching areas of consideration are:

• Ethical review of provenance and sourcing of the material
• Legality of collection, transfer and use (national and international)

Each transfer of samples is further undertaken according to a Research Collaboration Agreement or Material Transfer Agreement entered into by the Darwin Tree of Life Partner, Genome Research Limited (operating as the Wellcome Sanger Institute), and in some circumstances, other Darwin Tree of Life collaborators.

## Data Availability

European Nucleotide Archive: Lucilia caesar (common greenbottle). Accession number PRJEB83539. The genome sequence is released openly for reuse. The *Lucilia caesar* genome sequencing initiative is part of the Darwin Tree of Life Project (PRJEB40665) and the Sanger Institute Tree of Life Programme (PRJEB43745). All raw sequence data and the assembly have been deposited in INSDC databases. The genome will be annotated using available RNA-Seq data and presented through the Ensembl pipeline at the European Bioinformatics Institute. Raw data and assembly accession identifiers are reported in Tables 1 and 2. Production code used in genome assembly at the WSI Tree of Life is available at https://github.com/sanger-tol . Table 5 lists software versions used in this study.
