# Supplementary material for: The genome sequence of a greenbottle fly, Lucilia caesar (Linnaeus, 1758) (Diptera: Calliphoridae)
Source: Wellcome Open Res. 2026 Mar 17;11:163. [Version 1] doi: 10.12688/wellcomeopenres.26152.1 (PMC13125994; doi:10.12688/wellcomeopenres.26152.1)
Supplement: Peer review history

# Reviewer report: Reviewer response for version 1

**DOI:** 10.21956/wellcomeopenres.28799.r153283

**Published:** 2026-04-28

**Authors:** Jacqueline Heckenhauer

**Affiliations:** 1 Senckenberg Research Institute, Frankfurt, Germany

In this data note Falk et al. present the genome assembly of the greenbottle fly Lucilia caesar. The rationale for sequencing this species is clearly described: (1) the assembly was generated as part of the Darwin Tree of Life project, which produces reference genomes for species found in Britain and Ireland; (2) the species plays a role in forensic, medical and veterinary entomology, (3) the species is morphologically difficult to separate from other species of the genus, (4) molecular identification using DNA barcoding can be difficult; (5) the genome assembly will aid research on the phylogeny, taxonomy, biology and ecology of this interesting insect.

 The protocols used here are established and have been used for other DToL projects and all data is available.

Are sufficient details of methods and materials provided to allow replication by others?

Yes

Is the rationale for creating the dataset(s) clearly described?

Yes

Are the datasets clearly presented in a useable and accessible format?

Yes

Are the protocols appropriate and is the work technically sound?

Yes

Reviewer Expertise:

evolution, insects, silk, biodiversity genomics

I confirm that I have read this submission and believe that I have an appropriate level of expertise to confirm that it is of an acceptable scientific standard.

# Reviewer report: Reviewer response for version 1

**DOI:** 10.21956/wellcomeopenres.28799.r150989

**Published:** 2026-04-21

**Authors:** Rakesh K Mishra

**Affiliations:** 1 Tata Institute for Genetics and Society, Bengaluru, Karnataka, India

This manuscript describes the genome assembly of the greenbottle fly, Lucilia Caesar. It is a high-quality and useful contribution. The work utilized a robust multi-platform approach, including PacBio HiFi long reads for initial assembly, Hi-C for scaffolding, and RNA-seq for supporting future annotation.

 The genome assembly achieved a consensus quality (QV) score of 52.5, that exceeds the Earth BioGenome Project (EBP) benchmark, indicating a high level of base-pair accuracy in the final sequence. The analysis also identified 99.2% of expected genes, capturing nearly the entire protein-coding part of the genome of the species. Assigning the genome into six chromosomes, which is confirmed by Hi-C data provides high confidence in the large-scale structure of the genome. Lack of read for a Y chromosome, as authors speculate, may be due to XO male nature, a cytological or another technology can confirm.

 While the assembly is high-quality, it falls slightly below some EBP benchmarks. Additionally, a large number of centromeric repeats remain in unlocalised sequences. these, however, do not undermine the value of this work which may be helpful in addressing taxonomic challenges, forensic, medical/veterinary issues, not to mention its utility in evolutionary and ecological studies in future.

Are sufficient details of methods and materials provided to allow replication by others?

Yes

Is the rationale for creating the dataset(s) clearly described?

Yes

Are the datasets clearly presented in a useable and accessible format?

Yes

Are the protocols appropriate and is the work technically sound?

Yes

Reviewer Expertise:

Genome organization and eptiventic regulation

I confirm that I have read this submission and believe that I have an appropriate level of expertise to confirm that it is of an acceptable scientific standard.
